# Comparison of the Effectiveness of Greater Occipital Nerve (GON) Block and Sphenopalatine Ganglion (SPG) Block in the Treatment of Chronic Migraine

**DOI:** 10.3390/healthcare14142177

**Published:** 2026-07-19

**Authors:** Sara Kierońska-Siwak, Grzegorz Meder, Maciej Dzierżanowski, Jakub Wiśniewski, Magdalena Jabłońska, Dariusz Grzanka

**Affiliations:** 1 Department of Clinical Pathomorphology, Faculty of Medicine, Collegium Medicum in Bydgoszcz, Nicolaus Copernicus University in Torun, 85-094 Bydgoszcz, Poland; 2 Department of Interventional Radiology, Jan Biziel University Hospital No. 2, 85-168 Bydgoszcz, Poland; 3 Department of Physiotherapy, Faculty of Health Science, Collegium Medicum in Bydgoszcz Nicolaus Copernicus University in Toruń, 85-094 Bydgoszcz, Poland; 4 Department of Neurosurgery, Nicolaus Copernicus Hospital Gdansk, 80-803 Gdansk, Poland; 5 Doctoral School of Medical and Health Sciences, Collegium Medicum, Nicolaus Copernicus University, 85-094 Bydgoszcz, Poland

**Keywords:** chronic migraine, greater occipital nerve block, sphenopalatine ganglion block, interventional pain management, headaches

## Abstract

**Highlights:**

**What are the main findings?**

- Both greater occipital nerve (GON) block and sphenopalatine ganglion (SPG) block significantly reduced monthly migraine days, pain intensity, and migraine-related disability in patients with chronic migraine.
- GON block was associated with a greater reduction in monthly migraine days compared with SPG block.

**What are the implications of the main findings?**

- Peripheral nerve blocks may serve as effective adjunctive treatment options for patients with chronic migraine who remain symptomatic despite standard pharmacological therapy.
- GON block may provide greater clinical benefit than SPG block in reducing migraine frequency, although larger prospective studies are needed to confirm these findings.

**Abstract:**

**Background/Objectives:** Chronic migraine is a disabling neurological disorder that significantly impairs quality of life. Interventional procedures, including greater occipital nerve (GON) block and sphenopalatine ganglion (SPG) block, are increasingly used as adjuncts to standard pharmacological treatment. The aim of this study was to compare the effectiveness of GON and SPG blocks in patients with chronic migraine. **Methods:** This retrospective observational study included 50 patients diagnosed with chronic migraine according to the International Classification of Headache Disorders, 3rd edition (ICHD-3). Patients were assigned to either a GON block group or an SPG block group. Monthly migraine days, pain intensity measured using the Visual Analog Scale (VAS), and migraine-related disability assessed with the Headache Impact Test-6 (HIT-6) and Migraine Disability Assessment (MIDAS) questionnaires were evaluated before treatment and after completion of two procedures. **Results:** Both GON and SPG blocks were associated with significant reductions in monthly migraine days, pain intensity, and disability scores. A greater reduction in monthly migraine days was observed in patients treated with GON block compared with those treated with SPG block. **Conclusions:** Both GON and SPG blocks may represent effective adjunctive treatment options for patients with chronic migraine. In this study, GON block was associated with a greater reduction in migraine frequency, although further prospective studies are needed to confirm these findings.

## 1. Introduction

Migraine is among the most common neurological disorders and represents a major public health problem worldwide. It is estimated to occur in approximately 12–15% of the general population, with a markedly higher prevalence in women than in men [1]. A particularly severe form of the disease is chronic migraine. According to the criteria of the International Classification of Headache Disorders (ICHD-3), this diagnosis is established when headaches occur on at least 15 days per month for more than three months, with at least eight of those days meeting the criteria for migraine [1,2,3]. Standard migraine treatment includes both acute therapy for headache attacks and preventive treatment. Acute management involves, among other options, the use of nonsteroidal anti-inflammatory drugs, triptans, and other analgesic agents [4].

Despite the availability of numerous therapeutic options, some patients do not achieve satisfactory symptom control. For this reason, increasing attention has been directed in recent years toward interventional methods used in the treatment of headaches. These procedures may serve as an adjunct to standard pharmacotherapy and are particularly considered in patients with an inadequate response to pharmacological treatment [4,5].

The role of greater occipital nerve (GON) block in migraine treatment

The greater occipital nerve (GON) plays an important role in the pathophysiology of headaches, including migraine. It originates from the dorsal ramus of the second spinal nerve (C2) and is responsible for the sensory innervation of the posterior scalp. In the context of migraine, particular importance is attributed to the convergence of sensory fibers from the cervical spine and the trigeminal nerve within the so-called trigeminocervical complex [6]. This structure constitutes an important center for the integration of pain signals arising both from the facial region and the posterior part of the head. Excessive neuronal activity within this complex is believed to contribute to the persistence of chronic headache and to the phenomenon of central sensitization observed in patients with chronic migraine [5,7,8].

Greater occipital nerve block involves the administration of a local anesthetic in the region of the nerve’s course, resulting in temporary inhibition of pain signal transmission. However, the therapeutic effect of this procedure is not limited solely to peripheral action. Numerous studies suggest that GON block may lead to modulation of neuronal activity within the trigeminocervical complex, thereby reducing central nervous system hypersensitivity. Consequently, this may result in a decreased frequency of migraine attacks, reduced pain intensity, and limitation of cutaneous allodynia [9,10,11]. In clinical practice, greater occipital nerve block is most commonly used as an adjunctive treatment in patients with chronic migraine who remain symptomatic despite optimized pharmacological therapy. It may be used as a transitional treatment while preventive medications become effective or as an additional option in patients with inadequate response to preventive therapy. The procedure can be repeated at intervals of several weeks depending on the clinical response and recurrence of symptoms.

The role of sphenopalatine ganglion (SPG) block in migraine treatment

The sphenopalatine ganglion (SPG) is one of the most important autonomic ganglia within the head and plays a significant role in the regulation of the parasympathetic nervous system. It is located in the pterygopalatine fossa and constitutes an important component of the trigeminal–autonomic reflex involved in the pathophysiology of many primary headache disorders, including migraine [12,13,14]. Activation of parasympathetic fibers associated with this ganglion may lead to dilation of blood vessels within the meninges and to an exacerbation of neurogenic inflammation. Inhibition of impulses within the SPG may limit vasodilation and reduce the release of inflammatory neuropeptides such as substance P and CGRP [15,16]. As a result, this may lead to a reduction in pain intensity and shortening of the duration of migraine attacks [9,17]. Similarly, sphenopalatine ganglion block is used as an adjunctive interventional treatment in selected patients with chronic migraine who have persistent symptoms despite standard pharmacological management. It is particularly considered in patients with prominent cranial autonomic symptoms or when rapid symptomatic improvement is desirable. Repeated procedures may be performed depending on clinical response [12,13].

Because both GON block and SPG block are used as adjunctive interventional treatments for patients with chronic migraine refractory to standard pharmacological therapy, direct comparison of their clinical effectiveness may help optimize treatment selection in routine clinical practice. The aim of the present study was to compare the effectiveness of GON block and SPG block in patients with chronic migraine.

## 2. Materials and Methods

The analysis included 50 patients diagnosed with chronic migraine according to the International Classification of Headache Disorders (ICHD-3) criteria. Inclusion criteria comprised age ≥ 18 years, history of chronic migraine for at least 12 months prior to enrollment, persistence of symptoms despite standard pharmacological treatment (preventive pharmacotherapy included beta-blockers, topiramate, valproate, antidepressants and CGRP-targeted therapies when clinically indicated), stable preventive migraine therapy for at least 3 months before study initiation, and availability of complete clinical documentation, including follow-up assessments after the performed procedures.

Migraine frequency was obtained retrospectively from routine headache diaries documented in the medical records.

Exclusion criteria included secondary headache disorders, active infection at the planned injection site, known hypersensitivity to lidocaine or other local anesthetics, coagulation disorders or current anticoagulant therapy, pregnancy or breastfeeding, severe psychiatric disorders that could interfere with reliable symptom assessment, previous interventional headache procedures within the last 6 months, changes in preventive migraine pharmacotherapy during the observation period, and incomplete medical documentation or lack of follow-up data.

This retrospective study included consecutive adult patients treated in a single outpatient pain clinic.

The choice of intervention reflected routine clinical practice and was based on the treating neurosurgeon’s clinical judgment, taking into account headache characteristics, predominant pain location, cranial autonomic symptoms, previous treatment response, anatomical considerations and patient preference.

The primary endpoint was the between-group difference in change in monthly migraine days from baseline to two weeks after the second block.

### 2.1. Patient Characteristics

Patients were divided into two groups of 25 individuals each. In the first group, greater occipital nerve (GON) block was performed, whereas in the second group, sphenopalatine ganglion (SPG) block was administered. The mean age of the patients was 41.3 ± 10.8 years. Women constituted the majority of the study population. In the GON group, there were 20 women (80%) and 5 men (20%), whereas in the SPG group, there were 19 women (76%) and 6 men (24%). The mean disease duration from the time of diagnosis was 8.6 ± 4.2 years. No statistically significant differences were found between the groups with regard to age, sex, or disease duration (Table 1 and Table 2).

All patients remained on standard migraine treatment. Pharmacotherapy included both acute treatment of pain attacks and preventive therapy in accordance with current guidelines. No changes were introduced to pharmacological treatment during the study, and the medications used were not modified between the performed blocks.

### 2.2. Procedural Interventions

#### 2.2.1. Greater Occipital Nerve Block

Bilateral GON block was performed under ultrasound guidance by administering 4 mL of 2% lidocaine in the region of the nerve’s course.

#### 2.2.2. Sphenopalatine Ganglion Block

Bilateral SPG block was performed using a transnasal technique with an applicator, administering 2 mL of 4% lidocaine into each nostril.

In both study groups, the blocks were performed twice at intervals of 6–7 weeks. All GON and SPG blocks were administered bilaterally by a single experienced neurosurgeon to maintain procedural standardization throughout the study.

Different lidocaine concentrations were used because they reflect standard clinical practice for each intervention. Ultrasound-guided GON block is routinely performed using injectable 2% lidocaine, whereas transnasal SPG block typically requires 4% lidocaine to facilitate adequate mucosal penetration and diffusion to the ganglion. Therefore, the concentrations were selected according to established procedural protocols rather than to enhance treatment efficacy or influence comparative outcomes.

#### 2.2.3. Assessment of Treatment Effectiveness

The following parameters were analyzed: the number of migraine days per month, pain intensity on the VAS, HIT-6 score, and MIDAS score.

Assessments were carried out before the initiation of treatment and two weeks after the second block had been performed.

### 2.3. Statistical Analysis

Statistical analysis was performed using IBM SPSS Statistics version 28.0 (IBM Corp., Armonk, NY, USA). Continuous variables were presented as mean ± standard deviation (SD), whereas categorical variables were expressed as numbers and percentages. The normality of data distribution was assessed using the Shapiro–Wilk test.

To compare clinical parameters before treatment and after the administration of the blocks, the paired Student’s *t*-test was used or the Wilcoxon test in cases of non-normal data distribution. Differences between the GON and SPG groups were analyzed using the independent Student’s *t*-test or the Mann–Whitney test.

A *p*-value of <0.05 was considered statistically significant.

## 3. Results

### 3.1. Number of Migraine Days

Before the initiation of treatment, the mean number of migraine days was 18.4 ± 3.1 days/month in the GON group and 17.9 ± 3.4 days/month in the SPG group, with no significant difference between the groups (*p* = 0.62). After two blocks had been performed, a significant reduction in the number of migraine days was observed in both groups: to 9.8 ± 3.7 days/month in the GON group and 11.6 ± 4.1 days/month in the SPG group (*p* < 0.001). The mean reduction was 8.6 ± 2.1 days in the GON group and 6.3 ± 1.3 days in the SPG group (between-group *p* = 0.041).

**Figure 1:**
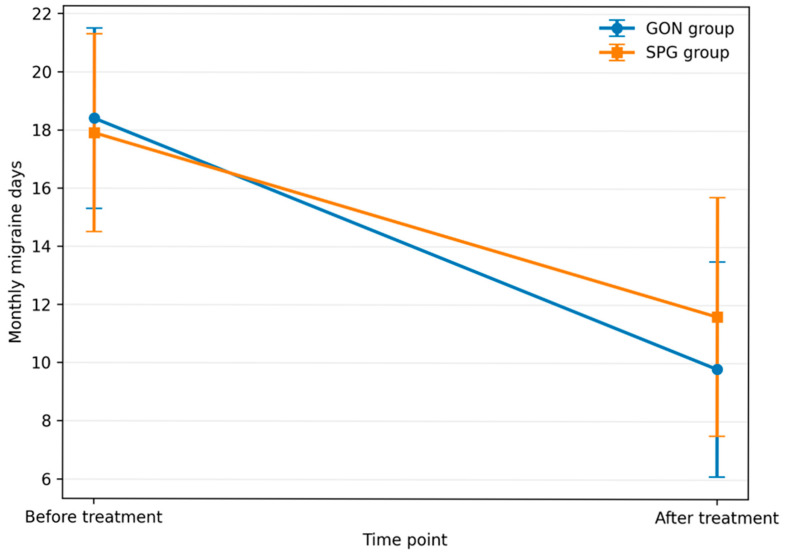
Mean monthly migraine days before treatment (baseline) and two weeks after the second intervention (post-treatment) in patients treated with greater occipital nerve (GON) block and sphenopalatine ganglion (SPG) block. Error bars represent standard deviations.

The reduction in the number of migraine days was greater in the GON group (*p* = 0.041). Mean monthly migraine days before treatmen and two weeks after the second intervention was presented in Figure 1.

### 3.2. Pain Intensity on the VAS

The mean pain intensity before treatment was 7.5 ± 1.2 in the GON group and 7.3 ± 1.1 in the SPG group (*p* = 0.48). At the end of the follow-up period, these values decreased to 4.2 ± 1.4 and 4.8 ± 1.6, respectively (*p* < 0.001 for both groups). A tendency toward greater pain reduction was observed in the GON group; however, the difference between the groups was not statistically significant (*p* = 0.09).

### 3.3. HIT-6 Scores

Before treatment, the mean HIT-6 score was 65.2 ± 5.1 in the GON group and 64.8 ± 5.4 in the SPG group (*p* = 0.74). After treatment, the values decreased to 56.3 ± 6.2 and 58.1 ± 6.7, respectively (*p* < 0.001). No significant differences between the groups were observed after treatment (*p* = 0.21).

### 3.4. MIDAS Scores

The mean MIDAS score decreased from 42.5 ± 12.3 to 22.8 ± 10.4 in the GON group and from 40.7 ± 11.8 to 26.1 ± 11.3 in the SPG group (both *p* < 0.001 for within-group comparisons). Although the reduction was numerically greater in the GON group, the between-group difference did not reach statistical significance (*p* = 0.27).

Changes from baseline are summarized in Table 3. The reduction in monthly migraine days was significantly greater in the GON group, whereas changes in VAS, HIT-6, and MIDAS scores did not differ significantly between groups.

### 3.5. Adverse Events

In the GON group, discomfort at the injection site was reported by 5 patients (20%), whereas transient occipital numbness occurred in 4 patients (16%). In the SPG group, transient nasal mucosal irritation was reported by 8 patients (32%). No serious adverse events or procedure-related complications were observed in either group.

## 4. Discussion

In the analyzed population, a greater reduction in the number of migraine days was observed in the group of patients who underwent greater occipital nerve block. This finding is consistent with reports from previous clinical studies. In a randomized study conducted by Inan et al., repeated GON blocks in patients with chronic migraine were shown to result in a significant reduction in the number of headache days and a decrease in pain intensity compared with the control group [18]. Similar observations were reported by Ashkenazi et al., who demonstrated in their studies that GON block may lead to a reduction in the frequency of migraine attacks and a decrease in pain intensity in patients with migraine resistant to pharmacological treatment [10]. Likewise, Dilli et al., in a study involving patients with chronic migraine, confirmed the beneficial effect of this procedure, observing a reduction in the number of headache days and improvement in quality-of-life parameters [9].

The mechanism of action of GON block is most commonly explained by modulation of the activity of the trigeminocervical complex. Within this structure, convergence occurs between sensory fibers originating from both the trigeminal nerve and the cervical segments of the spinal cord. As indicated by neurophysiological studies, inhibition of afferent sensory stimulation from the occipital nerve region may lead to a reduction in excessive neuronal activity within this complex and to attenuation of the phenomenon of central sensitization, which plays an important role in the pathophysiology of chronic migraine. This phenomenon may explain the observed reduction in the frequency of migraine attacks following GON block [19,20].

In contrast, sphenopalatine ganglion block affects a different component of the pathophysiology of migraine pain. The SPG constitutes an important element of the parasympathetic system within the head and is involved in the regulation of the trigeminal–autonomic reflex [14,19]. Activation of parasympathetic fibers may lead to dilation of meningeal vessels and increased release of inflammatory mediators such as CGRP or substance P. SPG block may limit these processes by inhibiting autonomic activity and reducing the release of inflammatory mediators. In a study conducted by Cady et al., transnasal SPG block was shown to result in a significant reduction in pain intensity in patients with migraine. Tepper et al. also indicated that modulation of sphenopalatine ganglion activity may constitute an effective treatment method for selected types of headache disorders, including migraine [21,22,23].

The observed differences between the GON and SPG groups should, however, be interpreted with caution. Because treatment allocation reflected routine clinical practice and was based on the treating physician’s clinical judgment rather than randomization, selection bias cannot be excluded. Although baseline demographic characteristics were comparable between groups, unmeasured clinical variables such as headache phenotype, predominant pain location, cranial autonomic symptoms, or previous response to treatment may have influenced treatment allocation and clinical outcomes. Therefore, the comparative findings should be interpreted cautiously.

The literature also emphasizes the importance of repeated blocks in achieving a more sustained therapeutic effect. Clinical studies on GON block indicate that a series of repeated procedures may lead to longer-lasting symptom reduction compared with a single block. Similar observations apply to SPG block, which in some studies was performed repeatedly at specific time intervals in order to maintain the therapeutic effect [24,25].

An important aspect of the present study was the maintenance of stable pharmacotherapy in all patients. No changes in pharmacological treatment were introduced during the observation period, and the preventive and acute medications used were not modified between the performed blocks. This made it possible to limit the impact of pharmacotherapy changes on the obtained results and to provide a more reliable assessment of the effectiveness of the interventional procedures themselves. In clinical practice, peripheral nerve blocks are most commonly used as part of combination therapy; therefore, evaluating their effectiveness under conditions of stable pharmacological treatment has significant practical relevance [26].

### Limitations

However, it should be emphasized that the present study has several limitations. First, the sample size was relatively small, and the study was not randomized. Because of the limited number of participants, the study may have been underpowered to detect statistically significant differences in secondary outcomes such as VAS, HIT-6, and MIDAS scores. Consequently, the absence of statistically significant between-group differences for these parameters should be interpreted with caution. Furthermore, the non-randomized treatment allocation may have introduced selection bias, limiting the ability to draw causal conclusions regarding the comparative effectiveness of the two interventions.

In addition, information regarding migraine phenotype, cranial autonomic symptoms, and predominant pain location was not systematically collected in the retrospective clinical records. Consequently, the potential influence of these clinical characteristics on treatment response could not be evaluated and should be addressed in future prospective studies.

Moreover, the follow-up period was relatively short and reflected only short-term clinical outcomes, precluding assessment of the long-term durability of treatment effects, safety, tolerability, and delayed adverse events. Future prospective studies involving larger patient populations, randomized treatment allocation, and longer follow-up periods are required to confirm the present findings.

## 5. Conclusions

The present findings suggest an association between GON block and a greater reduction in monthly migraine days compared with SPG block. However, because of the retrospective design, non-randomized treatment allocation and relatively small sample size, these observations should be considered exploratory and require confirmation in prospective randomized studies.

## Figures and Tables

**Table 1:** Baseline characteristics of the study population.

Parameter	GON Group (n = 25)	SPG Group (n = 25)	*p*-Value
Age (years), mean ± SD	41.7 ± 10.5	40.9 ± 11.2	0.78
Female sex, n (%)	20 (80%)	19 (76%)	0.73
Male sex, n (%)	5 (20%)	6 (24%)	0.73
Disease duration (years), mean ± SD	8.8 ± 4.1	8.4 ± 4.3	0.74
Migraine days/month, mean ± SD	18.4 ± 3.1	17.9 ± 3.4	0.62
VAS score, mean ± SD	7.5 ± 1.2	7.3 ± 1.1	0.48
HIT-6 score, mean ± SD	65.2 ± 5.1	64.8 ± 5.4	0.74
MIDAS score, mean ± SD	42.5 ± 12.3	40.7 ± 11.8	0.56

**Table 2:** Clinical outcomes before and after treatment.

Outcome Measure	Time Point	GON Group (n = 25)	SPG Group (n = 25)	*p*-Value Between Groups
Migraine days/month	Before treatment	18.4 ± 3.1	17.9 ± 3.4	0.62
	After treatment	9.8 ± 3.7	11.6 ± 4.1	0.041
VAS score	Before treatment	7.5 ± 1.2	7.3 ± 1.1	0.48
	After treatment	4.2 ± 1.4	4.8 ± 1.6	0.09
HIT-6 score	Before treatment	65.2 ± 5.1	64.8 ± 5.4	0.74
	After treatment	56.3 ± 6.2	58.1 ± 6.7	0.21
MIDAS score	Before treatment	42.5 ± 12.3	40.7 ± 11.8	0.56
	After treatment	22.8 ± 10.4	26.1 ± 11.3	0.27

**Table 3:** Change from baseline in clinical outcomes following greater occipital nerve (GON) block and sphenopalatine ganglion (SPG) block.

Outcome	Change from Baseline (GON)	Change from Baseline (SPG)	Between-Group *p*-Value
Monthly migraine days	−8.6 ± 2.1	−6.3 ± 1.3	0.041
VAS	−3.3 ± 1.5	−2.5 ± 1.6	0.09
HIT-6	−8.9 ± 4.2	−6.7 ± 4.5	0.21
MIDAS	−19.7 ± 9.8	−14.6 ± 10.1	0.27

## Data Availability

The data presented in this study are available from the corresponding author upon reasonable request. The data are not publicly available due to privacy and ethical restrictions.

## Abbreviations

The following abbreviations are used in this manuscript:

CGRP	Calcitonin Gene-Related Peptide
GON	Greater Occipital Nerve
HIT-6	Headache Impact Test-6
SPG	Sphenopalatine ganglion
